# Coccidia-Microbiota Interactions and Their Effects on the Host

**DOI:** 10.3389/fcimb.2021.751481

**Published:** 2021-10-01

**Authors:** Chenyang Lu, Yaqun Yan, Fuchun Jian, Changshen Ning

**Affiliations:** College of Veterinary Medicine, Henan Agricultural University, Zhengzhou, China

**Keywords:** coccidia, gut microbiota, host, interaction, probiotics

## Abstract

As a common parasitic disease in animals, coccidiosis substantially affects the health of the host, even in the absence of clinical symptoms and intestinal tract colonization. Gut microbiota is an important part of organisms and is closely related to the parasite and host. Parasitic infections often have adverse effects on the host, and their pathogenic effects are related to the parasite species, parasitic site and host-parasite interactions. Coccidia-microbiota-host interactions represent a complex network in which changes in one link may affect the other two factors. Furthermore, coccidia-microbiota interactions are not well understood and require further research. Here, we discuss the mechanisms by which coccidia interact directly or indirectly with the gut microbiota and the effects on the host. Understanding the mechanisms underlying coccidia-microbiota-host interactions is important to identify new probiotic strategies for the prevention and control of coccidiosis.

## Introduction

Coccidiosis is a self-limiting protozoal disease mainly caused by coccidia of the genus *Eimeria* (Kemp et al., 2013). *Eimeria* species are generally gastrointestinal parasites that cause different degrees of enteritis, such as diarrhea, dehydration, and weight loss. *Eimeria* is a large genus, with over 1,800 species identified to date (Duszynski, 2001). Compared with other genera and species related to coccidia, their life cycles are completed in a single host, and they have high host specificity. Generally, *Eimeria* are supposed not to spread between different host taxa (Bangoura and Bardsley, 2000), however, several of them are demonstrated to be able to infect among various species (Mácová et al., 2018; Trefancová et al., 2021). Furthermore, this genus has a highly diverse host range and affects all vertebrates (Duszynski, 2001).

All members of coccidia replicate and produce oocysts in the intestine of the final host, which enter into the environment with feces. Animals ingest sporulated oocysts from contaminated environments, which are transported to the intestine and then released as sporozoites (Chapman, 1978). Each sporozoite invades epithelial cells and remains within the parasitophorous vacuole during its development into trophozoites. The trophozoites begin asexual replication, at which point the parasite is referred to as a schizont. Each schizont forms thousands of first-generation merozoites. After a schizogony cycle is completed, the host cells are destroyed, and merozoites enter the intestinal lumen, where they infect new epithelial cells. After several generations of merogony, the parasite enters sexual replication, forming the dimorphic stages of macrogametes and microgametes. Microgametes enter the new host cell and fertilize the macrogametes to produce zygotes (Ferguson et al., 2003). After the zygote becomes an oocyst, it is released into the environment with feces (Shirley et al., 2005). Coccidia perform a series of life activities in the intestine of the host, including colonization, growth and reproduction, thereby disrupting the balance of the intestinal environment.

However, the mechanisms by which coccidia infect the organism and cause pathogenesis remain unknown. Most studies have focused on the pathogenesis of coccidia, mainly involving disruption of the intestinal mucosa and immunity. The gut microbiome is a complex network of symbiotic microorganisms with several functions that are beneficial to the host, including the absorption of nutrients, synthesis of essential organic compounds, protection from pathogens and development of the intestinal immune system. Coccidia and intestinal microbiota share an intestinal microenvironment. The composition of the gut microbiota is altered directly or indirectly *via* changes in the physiological characteristics, permeability, and antimicrobial peptide production in the intestine (Zaiss and Harris, 2016). In addition, alterations in the gut microbiota affect the colonization of the parasite in the host, infection status, and treatment of parasitic diseases (White et al., 2018). Therefore, this article describes the mechanisms underlying coccidia-microbiota-host interactions.

*Eimeria* species that cooperatively infect animals are usually referred to as coccidia based on the name of the group of unicellular parasites to which they belong. Although *Cryptosporidium* was formerly supposed to be closely related to coccidia, it now belongs to Gregarinasina (Adl et al., 2019); therefore it is described separately. This review concerns only *Eimeria* species.

## Interactions Between Coccidia and Gut Microbiota

The intestinal mucosal interface is a large and complex three-dimensional defense system composed of mechanical, biological, chemical, and immune barriers. The function of the mucosal barrier is to prevent harmful substances from entering the systemic circulation. The number of intestinal microorganisms in animals is approximately 10 times the number of cells in the body, forming an interdependent and interactive micro-ecosystem. The source of gut microbiota in livestock is similar to that of humans. The sheep intestine is first colonized by *Butyricicoccus* and Lachnospiraceae, followed by *Clostridiales*, *Lactobacillus*, and Ruminococcaceae (Zhuang et al., 2020). In various stages of sheep development, the intestinal microorganisms mainly include *Bacteroides*, *Lactobacillus*, and *Ruminococcus*, similar to goats (Li et al., 2019), piglets (Kim et al., 2011), and calves (Dias et al., 2018). The main gut microbiomes in human are *Actinobacteria*, *Bacteriodetes*, *Firmicutes*, *Proteobacteria* (Davenport et al., 2017). The source of the initial gut microbiota is different between poultry and mammals, and Firmicutes is the main phylum in poultry intestines. Actinobacteria, *Bacteroides*, Proteobacteria and others have also been reported (Waite and Taylor, 2015). Coccidial infection affects the composition of the host’s gut microbiota directly or indirectly, and changes in the gut microbiota may also influence the infectivity of coccidia.

Infection with coccidia significantly decreases bacterial diversity in the small intestine. In chickens, *Eimeria tenella* is the most pathogenic *Eimeria* species. Animals infected with *E. tenella* showed a reduced abundance of most bacterial taxa, except for members of the family Enterobacteriaceae. (Kimura et al., 1976). *E. tenella* infection enriches hostile bacteria, including *Bacillus*, *Enterococcus*, *Escherichia*, *Shigella*, *Staphylococcus*, and others. Furthermore, *Klebsiella*, and *Proteus* were also enriched (Cui et al., 2017). It was previously observed that *Eubacterium*, *Lactobacillus*, and *Ruminococcus* were significantly decreased in the caecum of broiler chickens orally challenged with oocysts of *Eimeria acervulina*, *Eimeria maxima*, and *Eimeria brunetti* (Stanley et al., 2014) and the ileum of broiler chickens inoculated with *E. maxima* (Kim et al., 2015). Furthermore, the infection greatly decreased the frequency of the immune-modulating bacterium *Candidatus arthromitus*. In a similar study, Ruminococcaceae members were reduced, and three unknown *Clostridium* species were increased after infection with these three *Eimeria* species (Wu et al., 2014). Our previous work assessed the gut microbiota of Hu sheep naturally and artificially infected with coccidia and found that infection caused an increase in Firmicutes and Proteobacteria and a decrease in *Bacteroidetes* and *Roseburia*. The study also showed that coccidial infection had a greater effect on the gut microbiota of lactating lambs, causing a significant decrease in Christensenellaceae and *Bifidobacteria* (Zhou, 2020). This finding suggests that coccidial infection may cause more severe disorders in young sheep. In summary, coccidial infection dramatically decreased resident microbiome and enriched a large number of conditionally pathogenic bacteria. In contrast, the abundances of probiotics, including *Alistipes*, *Blautia*, *Desulfovibrio*, Lachnospiraceae, *Lactobacillus*, *Roseburia*, and *Ruminococcus*, were reduced in coccidia-infected mice (Huang et al., 2018).

### Direct Interactions

The microbiome comprises bacteria, viruses, fungi, protozoa, and parasites, their comprehensive commensal, symbiotic, pathogenic, or parasitic relationship is important for health (Desselberger, 2018). The coexistence of microbiome and coccidia in the gut provides ample opportunities to interact with each other, both positive and negative (Leung et al., 2018). For example, supernatants of *Lactobacillus* had the inhibitory effects on the *E. tenella* (Tierney et al., 2004), meaning that some gut microbes have the capacity to directly attack sympatric coccidia. Certain probiotic bacteria have antimicrobial effects through their phagocytic antagonism and *via* the metabolism of acetic acid and other substances with broad-spectrum antimicrobial activity, which facilitates the inhibition of conditionally pathogenic bacteria (Biggs and Parsons, 2008). Now we have no evidence to demonstrate the mechanism about how the bacteria facilitate coccidia, while some research claimed that phagocytosis of pathogenic bacteria by *Entamoeba histolytica* induced virulence of parasite (Galván-Moroyoqui et al., 2008). Gaboriaud et al. (2021) compared the development of *E. tenella* in germ-free and conventional chickens, they observed the lower load of oocysts and the longer asexual phase in the absence of microbiota. Most likely this is because the digestive content and synthetizes metabolites synthetized by microbiota are crucial for the replication of coccidia (Gaboriaud et al., 2021). So it is important to identify the precise metabolites, and modulate the composition of the microbiota to inhibit the coccidia. Parasites and gut microbes may also interact by competing for the same nutrients or overlapping resource requirements. Following infection by coccidia, the balance between the organism and the microbiome is disrupted, resulting in dysbiosis of the gut microbiota. However, supplementation with beneficial microbiota protect against infection by competing with coccidia for space and resources (Butel, 2014).

### Indirect Interactions

#### Interactions With the Intestinal Mechanical Barrier

Tight junctions play a crucial role in maintaining the intestinal epithelial cell barrier, protecting the host intestine from pathogens and preventing the transmission of macromolecules (Schneeberger and Lynch, 2004). Tight junction-related proteins include occludin, zonula occludens, and claudins. *Eimeria vermiformis*-infection inhibits the epithelial cell mRNA expression of zonula occludens-1 in mice (Farid et al., 2008), and zonula occludens-1 downregulation or reduced activity affects the formation of intercellular tight junctions. With higher concentrations of coccidia, the expression of tight junction proteins was dose-dependently upregulated, with a simultaneous increase in gastrointestinal permeability, indicating more severe intestinal damage (Teng et al., 2020). Combined with the disruption of the mucus layer, this damage profoundly alters the interactions between the host and its microflora, allowing for greater microbial contact with the epithelial barrier and even penetration across the interface. After treatment with probiotics, the expression levels of claudin-1 and zonula occludens-1 were increased in the *E. tenella*-infected chicken (Memon et al., 2020). Probiotics maintain tight junction integrity of intestinal epithelial cells, mainly through the bioactive substances produced by their metabolism, to protect against pathogenic bacteria-induced damage of intestinal epithelial cells. A mixture of *Bacillus subtilis* and *Saccharomyces cerevisiae* increased the expression of tight junction-associated proteins, such as occludin, claudin-2, and claudin-3, in broiler chickens (Rajput et al., 2013).

Due to the invasion and replication of coccidia, the host cells are under pressure, which may cause apoptosis. To grow and survive in host cells, coccidia inhibit apoptosis by regulating anti-apoptotic factors. In *Eimeria intestinalis*-infected rabbits, the percentage of apoptotic cells in the ileum was significantly higher compared with the control group (Abdel-Haleem et al., 2017). Before the development of second-generation schizonts is completed, *E. tenella* may directly activate the NF-κB pathway in host cells to further inhibit host cell apoptosis. After developmental completion, *E. tenella* prevent the expression of NF-κB response genes and further reduce the expression of the anti-apoptotic proteins Bcl-2 and Bcl-XL, thereby accelerating host cell apoptosis and promoting the release of merozoites (Del et al., 2004). During their early development, *E. tenella* inhibit pro-apoptotic proteins by inducing anti-apoptotic factors to protect their cells and ability to proliferate (Del et al., 2004). Using probiotics, including *B. subtilis*, *Clostridium butyricum*, and *Lactobacillus*, we observed upregulated Bax expression and downregulated Bcl-2 levels in the *E. tenella-*infected chicken (Memon et al., 2020). Zhang et al. (2015) demonstrated that *E. tenella* promoted the apoptosis of cecal epithelial cells *in vitro*, especially during the middle to late stages. The use of specific inhibitors significantly decreased DNA injury, apoptosis, and caspase-9 and caspase-3 activity in chick embryo cecal epithelial cells after *E. tenella* infection (Li et al., 2017). Most probiotics inhibit the NF-κB pathway by impairing epithelial cell protease function and preventing the degradation of NF-κB (IκB) negative regulators (Jiang et al., 2012). The induction of apoptosis may become a new direction in the treatment of coccidiosis. The use of probiotics during the early stage of coccidial infection promotes the apoptosis of intestinal epithelial cells and reduces coccidial colonization and development.

#### Interactions With the Intestinal Chemical Barrier

The chemical barrier of the intestine consists of mucin (MUC),antimicrobial peptides (AMPs), regenerating islet-derived protein 3, lysozymes, and other factors (Okumura and Takeda, 2017). *Eimeria* infection significantly downregulates the gene expression of *MUC2* and *MUC5ac* (Jiang et al., 2013), resulting in a decrease in the content of MUC in the mucus layer. This prevents mucus layer replenishment and further disrupts the integrity of the intestinal mucosal chemical barrier. Mice infected with sporulated *Eimeria papillata* exhibit marked goblet cell hypoplasia and depleted mucus secretion (Dkhil et al., 2013). The number of colonic cup cells gradually decreases with the development of *Eimeria pragensis* endogenous life cycle stages (Yunus et al., 2005). Microorganisms, such as Actinobacteria, Bacteroidetes, Firmicutes, and Verrucomicrobia (Tailford et al., 2015), use mucus carbohydrates as a carbon source. Therefore, they may not gain a competitive advantage after a reduction in mucus production. It has been proposed that coccidia stimulate mucus production *in vivo*, leading to an increase in the relative abundance of MUC-utilizing bacteria, such as *Clostridiales* (Collier et al., 2008), whose growth *in vitro* was enhanced by the addition of MUC (Ramanan et al., 2016). The type and glycosylation of mucoproteins in the mucus layer covering the intestinal epithelium are different due to the various colonization sites of coccidia species in the intestine (Moncada et al., 2003), and both the MUC’s composition and glycosylation are known to affect the taxa that use the mucus (Sommer et al., 2014). Therefore, parasite-driven changes in mucus may alter the microbiota.

Host defense peptides exhibit direct antibacterial activity after coccidial infection and induce the expression of MUC and tight junctional proteins to enhance mucosal barrier function (Robinson et al., 2015). After infection with *Eimeria praecox*, several genes were downregulated, including those that encode antimicrobial peptide 2 and the cationic, anionic, and L-type amino acid transporters (Yin et al., 2015). Similar findings were reported for *E. maxima* (Casterlow et al., 2011) and *E. acervulina* (Su et al., 2014). *E. acervulina* and *E. maxima* challenge resulted in the downregulation of avian beta-defensin, which had antibacterial effects against *Actinobacillus*, *Candida albicans*, *Escherichia coli*, *Listeria monocytogenes*, and *Salmonella typhimurium* species (Elahi et al., 2005). The addition of moderate concentrations of quercetin to feed exerts a regulatory effect on the ileal avian beta-defensin and toll-like receptor (TLR) signaling pathways by reducing the abundance of *Clostridium* and increasing the levels of *Bifidobacterium*, thereby maintaining the ileal microecological balance and reducing mortality. In other words, increased host antimicrobial peptide production can improve the intestinal microbiota and subsequently ameliorate the symptoms of coccidia. *Lactobacillus* and some gram-positive bacteria enhance intestinal barrier function by inducing the NF-κB pathway and activating activator protein-1 and mitogen-activated protein kinase to upregulate β-defensin 2 (Schlee et al., 2008). Probiotics stimulate the host to produce active molecules, such as MUC and antimicrobial peptides, which may be one of their action mechanisms to enhance the body’s resistance to coccidial infection.

#### Interactions With the Immune System

Host anti-infectious strategies (including immune responses) are elicited following infection with parasites (Zhou et al., 2013). However, the immune system regulates the gut microbiota and their relative abundance to ensure a mutually beneficial host-microbe symbiosis. *Eimeria* species inhibit host immune responses to promote their invasion and colonization in hosts through negatively regulating the production of inflammatory cytokines (Zhao et al., 2018), thereby altering the gut microbiota.

The specific immune response to coccidiosis involves both cellular and humoral components. In infected animals, the humoral immune response indicates high titers of various antibody classes, beginning with the increase in IgM, followed by IgG, IgA, and others (Hughes et al., 1985). In an ovine model, increases in the IgG level and oocyst shedding occurred simultaneously during the primary infection and then decreased to baseline levels (Dalloul et al., 2005). Matos et al. (2018) demonstrated that *Eimeria ninakohlyakimovae* infected goats and revealed the increased levels of specific IgG, IgM, and IgA during the host immune response. By measuring the content of immunoglobulins and gut microbiota in inflammatory bowel disease patients, it was observed that IgG, IgM, and IgA had a positive correlation with Enterobacteriaceae and *Enterococcus*; while a negative correlation with *Lactobacillus* and *Bifidobacterium*. This indicates that IgM, IgG, and IgA are closely related to the imbalance in the gut microbiota, which may be caused by changes in the proportion and quantity of gut microbiota, leading to disruption of the intestinal mucosal microecological balance and abnormal immune responses. However, humoral immune reactions cannot eliminate primary coccidial infections (Daugschies and Najdrowski, 2005). Specific antibodies are reportedly produced in response to ruminant *Eimeria* infections, however, they are not protective. Although the specific mechanism of action by which intestinal IgA provides protection against coccidial infection remains unknown, it is hypothesized that IgA reduces the development of sporozoites or merozoites and prevents host cell invasion (Yun et al., 2000).

Although both cellular and humoral immunity are activated in response to coccidial infections (Daugschies and Najdrowski, 2005), several studies have shown that the cellular immune response mediated by T cells plays a key role in the protective immunity against coccidia. T-cell-mediated immune responses reduce the excretion of oocysts in animals infected with *Eimeria bovis* and mainly involve CD4^+^ and CD8^+^ lymphocytes (Sühwold et al., 2010). Matos et al. (2018) infected 3-, 4-, and 5-week-old goat kids with sporulated oocysts and subjected them to a homologous challenge 3 weeks later. The results demonstrated higher eosinophils and lymphocytes compared with challenged groups infected at 6, 7, and 8 weeks old. The activation of antigen-specific T cells from *Eimeria*-immune mice, cattle, and chickens has been demonstrated by lympho-proliferation assays (Lillehoj, 1986). In addition, the gut microbiota and its metabolites induce the differentiation of T cells by direct or indirect mechanisms, including T-bet^+^ Th1 cells, RORγt^+^ Th17 cells, Treg cells and GATA3^+^ Th2 cells (Lee and Kim, 2017), and coccidia colonization primarily mediates Th1 cell responses. *E. bovis*-mediated T cell activation was accompanied by increased levels of certain cytokines (such as IL2, IL4, and IFN-γ) known to participate in the regulation of complex networks, thereby activating the migration of immune cells to the site of infection (Taubert et al., 2008). *E. tenella* strongly induces an immune response and increases IL-8 and IL-6 expression in the cecum (Yu et al., 2020). Macrophages isolated from chickens infected with *E. tenalla* or *E. maxima* produced IL-1 *in vitro* and showed 80-fold increased mRNA levels of jejunal and cecum IL-1β after 7 days of culture. IL family members have a wide range of immunomodulatory functions and are highly beneficial for the host’s defense against coccidial infection. The administration of *B. subtilis* to chickens infected with coccidia increased the level of specific antibodies and regulated intestinal immunity by modulating the expression of IL-1β, IFN-γ, and CXCLi2 in the intestine (Lee et al., 2013). *Lactobacillus*-based feed products increased intestinal IFN-γ and IL-2 expression in chickens, resulting in a 14% reduction in fecal oocysts compared with the control group (Chaudhari et al., 2020).

**Figure 1:**
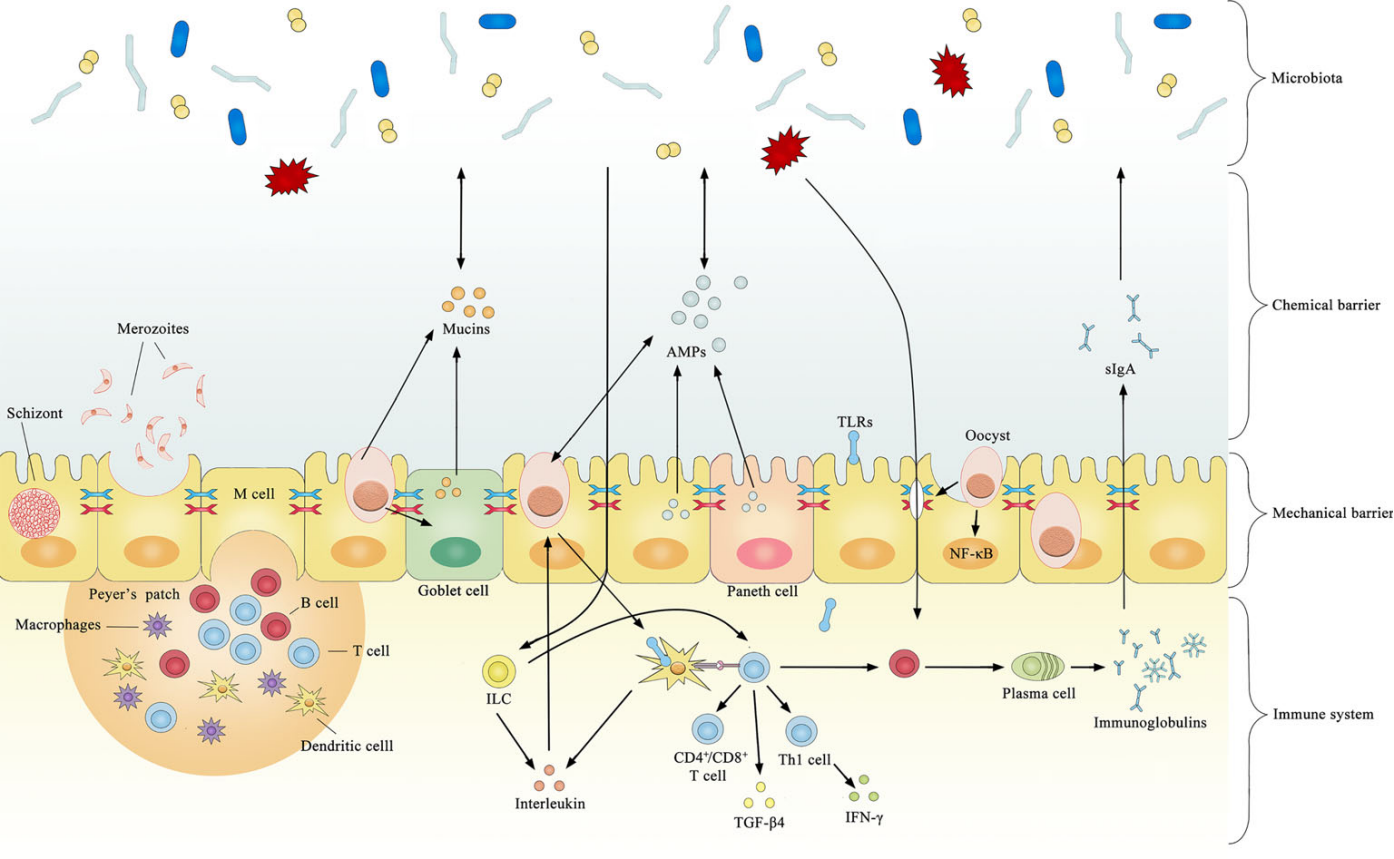
Summary of documented mechanisms by which infection with coccidia may indirectly interact with the gut microbiota.

Several cytokines are produced after coccidial infection, most of which have a coccidial suppressive effect *in vivo* or *in vitro*. However, some may have both pathological and immunophysiological effects. Significantly increased TLR2, TLR4, and TLR15 expression is observed after infection by coccidia (Zhou et al., 2013), and the upregulation of TLRs typically induces pro-inflammatory cytokines that regulate the immune response against bacterial infections. TLR2 mediates intestinal repair and barrier function to prevent pathogenic microorganism invasion by recognizing the cell wall components of gram-positive bacteria. In chickens, the expression level of TGF-β4 in intestinal intraepithelial lymphocytes was increased by 5- to 8-fold after coccidial infection (Jakowlew et al., 1997), and the expression of TGF-β4 in the spleen and cecum tonsils was increased by 3-fold (Song et al., 2010). The increased expression of TGF-β4 decreases the expression of IFN-γ, preventing excessive inflammation from causing damage to the organism. This may be a potential mechanism regulating mucosal inflammatory responses against intestinal microbes to maintain intestinal immune homeostasis. The current literature on immune-mediated interactions between coccidia and the microbiota is limited, and most previous studies focused on how microorganisms enhance immunity against coccidia without considering the opposite circumstance. For example, *Toxoplasma gondii* was found to induce TLR2, TLR4, and TLR9 signaling through the stimulation of gut microbiota and indirectly stimulate dendritic cells to activate innate and adaptive immune responses (Benson et al., 2009). In healthy organisms, the gut microbiota activates B cell receptors or TLRs to promote antigen presentation and antibody production (Buchta and Bishop, 2014). Collectively, these results suggest that coccidia and the microbiota have a complex relationship and interact across the mechanical barrier, chemical barrier and immune system (**Figure 1**).

## Impact of Coccidia-Microbiota Interactions on the Host

### Secondary Infection With Other Pathogens

#### Secondary Infection With Pathogenic Bacteria

Various studies have demonstrated the complex interactions of coccidia with bacteria, fungi, viruses or other intestinal parasites (Motha and Egerton, 1984; Fukata et al., 1984; Ruff and Rosenberger, 1985), which may lead to more severe clinical manifestations and economic losses. Changes in the gut microbiota caused by coccidial infection provide an environment that is conducive for the reproduction of pathogenic bacteria. Coccidial infections not only enhance the colonization of *Campylobacter jejuni* (Macdonald et al., 2019), *Clostridium perfringens* (Ficko-Blean et al., 2012), *Salmonella* (Kogut et al., 1994) and other bacteria but also increase their pathogenicity (Dykstra and Reid, 1978). This increases livestock and poultry diseases, thereby reducing animal performance, reproductive capacity and egg production and potentially leading to death. And coccidial infection causes a marked inflammatory response in the intestine, and the presence of inflammation favors the colonization of aerobic bacteria, especially Enterobacteriaceae (Lupp et al., 2007), which have been shown to exacerbate the increase in pathogenic bacteria. Enterobacteriaceae and *Lactobacillus* are antagonistic, and an increase in the number of Enterobacteriaceae may inhibit the intestinal colonization by *Lactobacillus* (Tortuero, 1973). A reduction in anaerobic bacteria in the intestine after coccidial infection in chickens was suggested to potentially decrease the concentration of volatile fatty acids in the cecum and induce changes in pH and oxidation-reduction potential in the intestine, which may directly lead to enhanced pathogenic infection (Qin et al., 1995). For example, a reduction in *Lactobacillus* after coccidial infection prevents the production of large amounts of lactic and acetic acid to effectively inhibit the invasion of *Salmonella enteritidis* (Bjerrum et al., 2006). The damage induced by coccidia appears to promote the spread and colonization of *C. perfringens* deep in the mucosa, and in some cases, this extends to the crypts and causes focal necrosis (Ficko-Blean et al., 2012), leading to secondary necrotic enteritis (Hofacre et al., 1998). The severity of necrotic enteritis has been reported to be associated with an increase in Proteobacteria and a decrease in Firmicutes (Xu et al., 2018), and these changes occurred during coccidial infection. Firmicutes were important for suppressing or eliminating *C. perfringens* and restoring intestinal homeostasis (Fasina et al., 2016). In addition, coccidial infections significantly increased Bacteroidetes, including Bacteroidaceae and Rikenellaceae. Bacteroidetes can damage intestinal epithelial cells and increase the invasion of other pathogens, thereby inducing or exacerbating enteritis. We speculate that the increase in Bacteroidetes and decrease in Firmicutes may be related to secondary infections with bacterial diseases.

#### Secondary Infections With Virus

Coccidial infection, which reduces the abundance of the microbes, is associated with low immunity. Virus-coccidial co-infection reportedly increased viral replication and delayed the clearance of viruses, such as avian leukosis virus (Cui et al., 2017), Marek’s disease virus (Biggs et al., 1968), infectious bursal disease virus (Giambrone et al., 1977), reticuloendotheliosis virus (Motha and Egerton, 1984) and reoviruses (Ruff and Rosenberger, 1985). Many conditionally pathogenic bacteria were significantly enriched in the intestine of coccidia-infected chickens, including Firmicutes and Proteobacteria. The significant enrichment of these conditionally pathogenic bacteria may be a key factor in the increased occurrence of secondary infections of avian leukosis virus (Dong et al., 2015). On the other hand, coccidia parasitize the intestinal epithelium and cause changes in the intestinal environment, like changes in metabolites such as SCFAs, which will influence the antiviral immune response (Chapman et al., 2013; Budden et al., 2017). *Subdoligranulum*, which decreases dramatically after coccidial infection, belongs to the subgroup of Clostridiales and is capable of butyrate production (Bjerrum et al., 2006). Butyrate reduces chronic inflammation by modulating the immune system, and its reduction may lead to increased chronic inflammation and immune disorders (Lund et al., 2010). Meanwhile, coccidial specific antigens can affect the activity of lymphocytes and suppress the immune response (Rose and Hesketh, 1984). When damaged the gut microbiota of chickens, we can observed higher cloacal and oropharyngeal shedding of avian influenza H9N2 in chickens, with the compromised type I IFNs and IL-22 expression (Yitbarek et al., 2018). So we may conclude that the coccidial infection may contribute the replication of virus. And dual infection of coccidia and virus will extend the replication time of the virus (Gao et al., 2015), which exacerbates clinical symptoms and leads the increased mortality (Giambrone et al., 1977).

### Impact on Host Metabolism and Nutrition

Short-chain fatty acids (SCFAs), which are the most widely and intensively studied end product of intestinal metabolism, play an important role in metabolism (Ley et al., 2006). These mainly include acetic acid, propionic acid, and butyric acid. The common SCFA-producing bacteria are mainly anaerobic bacteria, including *Bacillus*, *Bifidobacterium*, *Clostridium*, *Streptococcus*, and others (Garcia et al., 2008). However, following stimulation by the external environment and pathogenic microorganisms, the gut microbiota is severely damaged, leading to changes in the contents of SCFAs. This subsequently disrupts the metabolism of SCFAs, energy efficiency of food intake, and metabolic homeostasis of the body, resulting in the development of intestinal and metabolic diseases. The concentration of SCFAs in the intestine of animals infected with coccidia markedly changes. In particular, the concentration of acetic acid decreases, the levels of butyric and isovaleric acids increase, and the concentration of isobutyric acid increases or is unaffected (Stanley et al., 2014). Acetic acid has broad-spectrum antibacterial effects, acting as an inhibitor against *E. coli*, *Salmonella*, *Streptococcus*, and *Pseudomonas aeruginosa* in the intestine (Liévin et al., 2000). A reduction in acetic acid often leads to secondary infection with coccidia. Infection with *E. tenella* drastically reduces butyrate-producing *Subdoligranulum* in the cecum (Bjerrum et al., 2006). In addition, butyrate plays an important role in animal health by regulating the immune system and reducing chronic inflammation. Therefore, a decrease in butyrate may lead to a high prevalence of chronic inflammation and immune disorders. Upon coccidial infection, SCFAs are reduced, and the pH is increased in the cecum (Leung et al., 2019). SCFAs are known to reduce intestinal pH, which promotes the growth and proliferation of probiotic bacteria and inhibits the colonization of specific pathogenic bacteria. Furthermore, SCFAs are an important mediator of signal transmission from microbiota to host cells, including enteroendocrine, immune, and nerve cells (Rhee et al., 2009), which indirectly influences homeostasis in the intestinal lumen.

Coccidial infection affects the amount of nutrients in the body’s tissues by disrupting the normal gut microbiota. Damage to the mucosa also leads to impaired digestion because the gut microbiota is involved in protein metabolism. Food and endogenous proteins are hydrolyzed into peptides and amino acids by proteases and peptidases produced by the host and bacteria, releasing amino acids (Macfarlane et al., 1988). The digestion and absorption of proteins were shown to be impaired after infection with *Eimeria necatrix*, *Eimeria mitis*, and *E. maxima* (Turk, 1972). In contrast, protein uptake was increased at some time points after infection with *E. necatrix* or *E. acervulina* (Turk, 1972), and infection with *E. brunetti* had no effect (Fetterer et al., 2014). This may be due to the differences in their pathogenicity and the degree of disruption of the normal gut microbiota. In chickens infected with *E. acervulina*, the total plasma lipid level was significantly decreased (Allen, 1988). This may be caused by the reduced relative abundance of the dominant microbes following coccidial infection-induced increases in oxidative stress in the intestine, which promotes the secretion of reactive oxygen species from the intestinal epithelium. Excess reactive oxygen species directly targets DNA, lipids, and proteins in the cells of the organism, causing changes in their function and structure, which subsequently induces oxidative stress, decreases host food intake and impairs energy metabolism (Cooke et al., 2003). Coccidial infection also alters carbohydrate metabolism and uptake. Downregulated sucrase-isomaltase (SI) and glucose transporter 2 (GLUT2) were observed in the duodenum of *E. acervulina*-challenged animals (Su et al., 2014). It has been shown that the activity of SI in the small intestinal mucosa was inhibited in rats following disruption of the gut microbiota (Nanthakumar et al., 2013). In addition, reduced expression of SI and GLUT2 may lead to inhibition of the carbohydrate supply in tissues (Treem, 2012), thereby preventing body weight gain. The results from studies on blood glucose levels have been inconsistent, but most have found that coccidiosis leads to a significant decrease in blood glucose levels. Therefore, we conclude that the interaction between coccidia and the microbiota alters proteins, lipids, glucose, and other factors.

## Effects of Host Changes on Coccidia

The infection of animals with coccidia induces specific and long-term immune protection against coccidia and ameliorates the disruption of microbiota to a certain extent. It is generally accepted that coccidia has better immunogenicity in the early stages (endogamous stage) than in the later sexual stages. In coccidia-infected animals, the amount of sIgA is increased, which prevents microorganisms from residing and multiplying in the mucosal epithelium. sIgA can inhibit the invasion of bacteria in epithelial cells, increase the diversity of gut microbiota, and promote immune responses in intestinal epithelial cells (Hooper et al., 2012 and Mirpuri et al., 2014). IL-22 directly induces Reg IIIγ production in intestinal epithelial cells, thereby limiting the proliferation of *C. arthromitus*. The overgrowth of *C. arthromitus* not only increases the number of Th17 cells but also triggers Th17 cell-mediated intestinal inflammation, and T-bet expression in ILCs limits the accumulation of *Klebsiella pneumoniae*, and *Proteus mirabilis* to some extent (Kamada and Núñez, 2014). Reg IIIγ incubation with 10^5^~10^6^ CFU/mL *Listeria monocytogenes* or *Enterococcus faecalis* significantly decreases the bacterial survival rate and prevents the infection of the intestinal tract by pathogenic bacteria (Cash et al., 2006). The immune system regulates the structure of the intestinal microbiota through a variety of antimicrobial peptides secreted by intestinal epithelial cells, and defensins effectively kill several gram-positive and -negative bacteria, including *C. albicans, E. coli*, and *Enterococcus*, thereby restoring the normal microbial community composition.

The nutritional intake of the host also has a significant impact on the microflora composition and severity of coccidial infection. Similarly, the condition of the organism affects the infective ability of coccidia. Richter and Wiesner (1988) showed that increased levels of dietary crude protein from 11.3% to 12.4% reduced the mortality of chickens infected with coccidia by 16%. However, high protein contents were conducive to the development and reproduction of coccidia in the body. In addition, decreased dietary protein levels from 16% to 13% increased the abundance and diversity of ileal flora, including *Lactobacillus* and *Megasphaera*. In growing pigs, a 10% reduction in the protein level decreased the diversity of ileal and colonic flora (Fan et al., 2017). Therefore, the crude protein level in the diets of infected animals should not be too high or too low, and further research is necessary to determine the optimal diet composition.

## The Anti-Coccidial Application of Probiotics

In the past, the treatment of coccidiosis mainly involved anti-coccidial drugs, which inhibit the asexual and sexual reproduction stages of coccidia (Odden et al., 2018). For example, diclazuril is the most common chemistry medicine in the coccidial infection, which can be used to reverse the microbial changes induced by *Eimeria* spp. (Wang et al., 2021). However, some research treatment of enrofloxacin and diclazuril altered the abundance of gut microbiota and their functional metabolite pathways, reducing bacterial diversity while expanding and collapsing composition of specific indigenous microbes, than formed a new microbial community (Elokil et al., 2020). So we may conclude that long-term chemical treatment caused irreversible movement to gut microbiota although the drugs are effective to coccidia. Furthermore, the genetic diversity of *Eimeria* species contributed to the development of anticoccidial drug resistance, severely limiting the long-term disease prevention ability of these agents (Tan et al., 2017). Probiotics are a new type of anticoccidial drug that take advantage of the mutually antagonistic relationship between the gut microbiota and coccidia. To treat coccidial infection, probiotics may manipulate the gastrointestinal tract by restoring balance to the intestinal microbial community, improving intestinal tissue morphology and stimulating specific and non-specific immunity. Probiotics are classified as autochthonous microbiota, allochthonous microbiota, and fungus according to the source and action mechanism of the strain.

### The Function of Autochthonous Microbiota

Autochthonous microbiota come from the gut microbiota (Dubos et al., 1965), such as *Bifidobacterium*, *C. butyricum, Lactobacillus*, and *Streptococcus faecalis*. After obtaining the autochthonous microbiota, it can directly replenish the bacteria of origin and effectively colonize, reproduce and exert specific probiotic effects in animals (Mukai et al., 2002). The physiological and metabolic activities of autochthonous microbiota are closely related to the host. They can not only synthesize nutrients for the host and help maintain normal growth and life activities, but also form a biological barrier to prevent the invasion of pathogenic bacteria that compete for nutrients (Nava and Stappenbeck, 2011). In a previous study, the spent culture supernatant (SCS) of live and dead *Lactobacilli* was added to coccidia cultured *in vitro*, and the highest inhibition was found in the SCS of the live bacteria group, suggesting that the anticoccidial component is a secreted metabolite of lactic acid bacteria (Tierney et al., 2004). Exposure of *E. acervulina*, *E. tenella*, and *E. maxima* oocysts to the cell-free supernatant (corresponds to SCS) of *Lactobacillus rhamnosus* inhibited the sporulation of oocysts, which demonstrated the anti-coccidial activity of SCS (Biggs and Parsons, 2008). It has been shown that *Lactobacillus salivarius* produced antibacterial substances against *Brachyspira hyodysenteriae*, *C. jejuni*, *C. perfringens, E. coli*, and *Salmonella choleraesuis* (Klose et al., 2006). *C. butyricum* decreased the abundance of harmful bacteria, such as *Brachybacterium*, and *Candidatus arthromitus*, and increased the abundance of beneficial bacteria, such as *Lactobacillus* (Huang et al., 2019).

### The Function of Allochthonous Microbiota

Allochthonous microbiota, such as *Bacillus cereus*, *Bacillus licheniformis*, *B. subtilis*, are not closely related to the host, and they either colonize the digestive tract for a short period or do not colonize it at all (Bäckhed et al., 2005). Allochthonous and autochthonous microbiota have symbiotic effects whereby allochthonous microbiota promote the growth and multiplication of autochthonous microbiota (Bortoluzzi et al., 2019; Whelan et al., 2019). Autochthonous microbiota generally induce the production of low antibody levels in the host, whereas allochthonous microbiota induce a strong immune response (Guo et al., 2021). *B. subtilis* clearly elevated serum nitric oxide levels in coccidia-infected chickens (Lee et al., 2014). Nitric oxide induced sporozoites to escape before maturity, which inhibited coccidia reproduction (Yan et al., 2021). Nitric oxide-induced sporozoites significantly decreased the invasive ability and reproductivity in chickens compared with fresh sporozoites. In coccidia-infected chickens, feed containing *B. licheniformis* significantly increased the expression of IL-10 and JAM2 (Chaudhari et al., 2020). We conclude that *Bacillus* eliminate coccidia by increasing immune factors that induce sporozoite escape before maturity. *Bacillus* spp. produce an antimicrobial factor that inhibits the colonization of gram-positive pathogens, such as *B. cereus*, *Campylobacter coli*, *C. jejuni*, *Clostridium difficile*, *C. perfringens*, *L. monocytogenes*, *Micrococcus luteus*, *Staphylococcus aureus*, and *Streptococcus pneumoniae* (Khochamit et al., 2015).

### The Function of Fungus

Fungi commonly used include *S. cerevisiae* and *Saccharomyces boulardii*, which have specific mechanisms. In general, bacterial probiotics are generally sensitive to antibiotics. In contrast, fungal cell walls consist of two layers, forming a natural barrier. As a result, antibiotics cannot penetrate the cell wall to combine with nucleoproteins and interfere with the synthesis of nucleic acid, which makes yeast naturally resistant to antibiotics (Neut et al., 2017; Terciolo et al., 2019). Supplementation with *Saccharomyces* inhibited intestinal lesion formation and produced higher antibody titers (geomean titers), which provided protection against *Eimeria* infection in broilers (Awais et al., 2019). *Meyerozyma guilliermondii* isolated from chickens reduced *E. tenella* oocyst viability by damaging the resistant structure of oocysts, limiting their growth (Dantán-González et al., 2015). The action mode of yeasts in controlling intestinal diseases has not yet been elucidated, however, it is associated with the release of antimicrobial peptides, acidification of the surrounding environment, modification of inflammatory and immune responses and disruption of virulence factors (Hatoum et al., 2012). As immunomodulators, yeast cell wall components (β-glucans and mannans) are associated with immune system regulation, increasing local mucosal IgA secretion and cellular and humoral immune responses (Gómez-Verduzco et al., 2009). Dietary yeast cell wall (1 or 10 g/kg) reduced the severity of infection and oocyst shedding of a mixture of *E. acervulina*, *E. maxima*, and *E. tenella* (Elmusharaf et al., 2007) in broiler chickens. In addition, several investigations have shown that co-supplementation with yeast and bacterial probiotics improves survival and growth rates.

Several studies have confirmed the significant effect of probiotics on preventing coccidiosis, however, the exact mechanism has not yet been elucidated, and the following questions still need to be addressed. (1) How do probiotics regulate the gut microbiota to resist coccidia? (2) How do probiotics act on the intestinal biological barrier to exert anticoccidial effects? (3) What are the active ingredients of probiotics against coccidia? (4) Which probiotic has the best anticoccidial effect? In summary, a comprehensive understanding of the molecular mechanisms by which probiotics exert their beneficial effects on the host against coccidial infection is required for the development of highly effective probiotic formulations that can replace antibiotics for the prevention and control of coccidiosis.

## Conclusion

In recent years, with the development of high-throughput sequencing technology, research on the interrelationship between the gut microbiota and diseases has progressed, and an increasing number of researchers have recognized the important role of gut microbiota in disease onset, progression, treatment, and prognosis. Although the mechanisms by which coccidia and intestinal microbiota interact are not well understood, this review analyzed the different aspects of their interactions. Coccidia share the intestinal environment with microbiota and directly antagonize commensal bacteria. In addition, coccidia indirectly affect the intestinal microbiota. Mechanical mucosal damage (impaired tight junctions and apoptosis of intestinal epithelial cells), chemical mucosal damage (increased mucus production and decreased antimicrobial peptides) and disruption of the immune system provide conditions suitable for the growth of conditionally pathogenic bacteria, leading to changes in the intestinal microbiota. The addition of probiotics directly or indirectly impair coccidia development by improving the intestinal microbiota.

Coccidia-microbiota-host interactions form a network of mutual constraints. For example, coccidial infection causes an imbalance in the intestinal microbiota, which not only leads to a decrease in food intake and impaired absorption but also increases the susceptibility of the organism to secondary infections. Conversely, the disruption of host health increases the number of pathogenic bacteria and impairs the intestinal mucosal barrier function and the ability of the immune system to target coccidia, resulting in a more serious coccidial infection. Coccidia, the microbiota and the host simultaneously interact, and a change in one factor may affect the entire network.

In conclusion, a holistic approach is needed to gain a better understanding of the mechanisms underlying coccidia development and infection. However, most studies on coccidiosis have focused on avian species, with limited studies on ruminants. With the development of intestinal microbiota sequencing technology in recent years, we can improve our understanding of the mechanisms contributing to coccidia-microbiota-host interactions and provide a theoretical basis for the control of coccidiosis.

## Author Contributions

CL wrote the manuscript. CN and YY revised the manuscript. All authors read and approved the final version of the manuscript for publication.

## Funding

This work was supported by the Earmarked Fund for China Modern Agro-industry Technology Research System (No. nycytx-38) and National Key R&D Program of China (No. 2018YFD0502100).

## Conflict of Interest

The authors declare that the research was conducted in the absence of any commercial or financial relationships that could be construed as a potential conflict of interest.

## Publisher’s Note

All claims expressed in this article are solely those of the authors and do not necessarily represent those of their affiliated organizations, or those of the publisher, the editors and the reviewers. Any product that may be evaluated in this article, or claim that may be made by its manufacturer, is not guaranteed or endorsed by the publisher.
